# Development of a nomogram for screening of hepatitis B virus-associated hepatocellular carcinoma

**DOI:** 10.18632/oncotarget.22498

**Published:** 2017-11-18

**Authors:** Jung Wha Chung, Eun Sun Jang, Jaihwan Kim, Sook-Hyang Jeong, Nayoung Kim, Dong Ho Lee, Kyung Ho Lee, Jin-Wook Kim

**Affiliations:** ^1^ Department of Medicine, Seoul National University Bundang Hospital, Seongnam, Republic of Korea; ^2^ Department of Internal Medicine, Seoul National University College of Medicine, Seoul, Republic of Korea; ^3^ Department of Radiology, Seoul National University College of Medicine, Seoul, Republic of Korea

**Keywords:** chronic hepatitis B, hepatocellular carcinoma, nomograms, early diagnosis of cancer, ultrasonography

## Abstract

Current strategy of hepatocellular carcinoma (HCC) surveillance evaluates individual risks of HCC for defining candidates for surveillance, but estimated risks are not utilized for clinical decision-making during actual screening. We sought to determine whether consideration of individual risks improve the performance of ultrasound (US)-based HCC screening in a real-world chronic hepatitis B (CHB) cohort. This single center retrospective cohort study analyzed 27,722 screening US tests from 4,175 consecutive CHB patients. Logistic regression analysis was performed to identify independent parameters predicting presence of HCC. A nomogram was built based on the independent predictors of HCC and compared with US-only screening by receiver operating characteristics analysis. The cost-effectiveness of the nomogram was assessed by decision curve analysis. HCC developed in 222 patients with the incidence of 0.769 per 1000 person-year during the median follow-up of 63 months. Age, sex, presence of cirrhosis, serum alpha-fetoprotein (AFP) levels and positive US test results were independent predictors of HCC presence. A nomogram based on these predictors showed higher C-statistics compared to US-only screening (0.960 vs. 0.731 and 0.935 vs. 0.691 for derivation and validation cohort, respectively; *p* < 0.001). Decision curve analysis showed higher net benefit of the HCC nomogram-guided screening model compared to US-only screening in the risk threshold range between 0 and 0.3. A nomogram composed of age, sex, presence of cirrhosis, serum AFP levels and US findings better predicted the presence of HCC compared to US-only screening in CHB on surveillance.

## INTRODUCTION

Chronic hepatitis B virus infection is one of the leading causes of hepatocellular carcinoma (HCC) worldwide [1]. Surveillance for HCC is recommended for chronic hepatitis B (CHB) patients with increased risks [2–4], and ultrasonography (US) is a universally recommended screening test for HCC surveillance [3–5]. There have been concerns, however, about the sensitivity of screening US for small HCC in CHB, especially in the presence of regenerative nodules and fibrous septa [6–8]. Dynamic imaging techniques such as 4-phase multidetector computed tomography (CT) and dynamic contrast enhanced magnetic resonance imaging (MRI) have better sensitivity for small HCC compared to US [9], and the high specificity of dynamic imaging techniques obviate the need for biopsy when typical enhancing patterns are observed [10]. Considering the radiation hazards and high cost, however, dynamic imaging modalities are reserved for occasions when screening US suggests possibility of HCC [3, 5] or technical issues hamper optimal US evaluation [3, 11].

Bayesian theorem indicates that the post-test probability of a disease is determined by pre-test disease probability and likelihood ratio of the corresponding test [12, 13]. From the Bayesian perspective, the HCC probability of a CHB patient on surveillance is dependent not only on the results of the screening US but also on the baseline probability of HCC. Clinical and laboratory parameters such as age, sex, ethnicity, hepatitis B virus (HBV) viral loads, presence of cirrhosis and elevated alpha-fetoprotein (AFP) levels have been validated for predicting the risk for HCC incidence [14–19], and it is suggested that these parameters may also estimate the probability for immediate development of HCC [20]. Current guidelines employ these risk predictors in defining at-risk population for surveillance, but individual risks are not considered in the decision to implement an enhanced follow-up or to trigger a recall policy during surveillance [3–5].

We speculated that predictors of long-term HCC risk may also be used for estimating the probability of HCC presence, and that integrating these predictors may improve the accuracy of the US-based screening. To test this hypothesis, we developed a nomogram predicting presence of HCC in a real-world CHB cohort on surveillance, and compared the screening performance of the nomogram with that of traditional US-only screening.

## RESULTS

### Characteristics of study cohort

**Figure 1:**
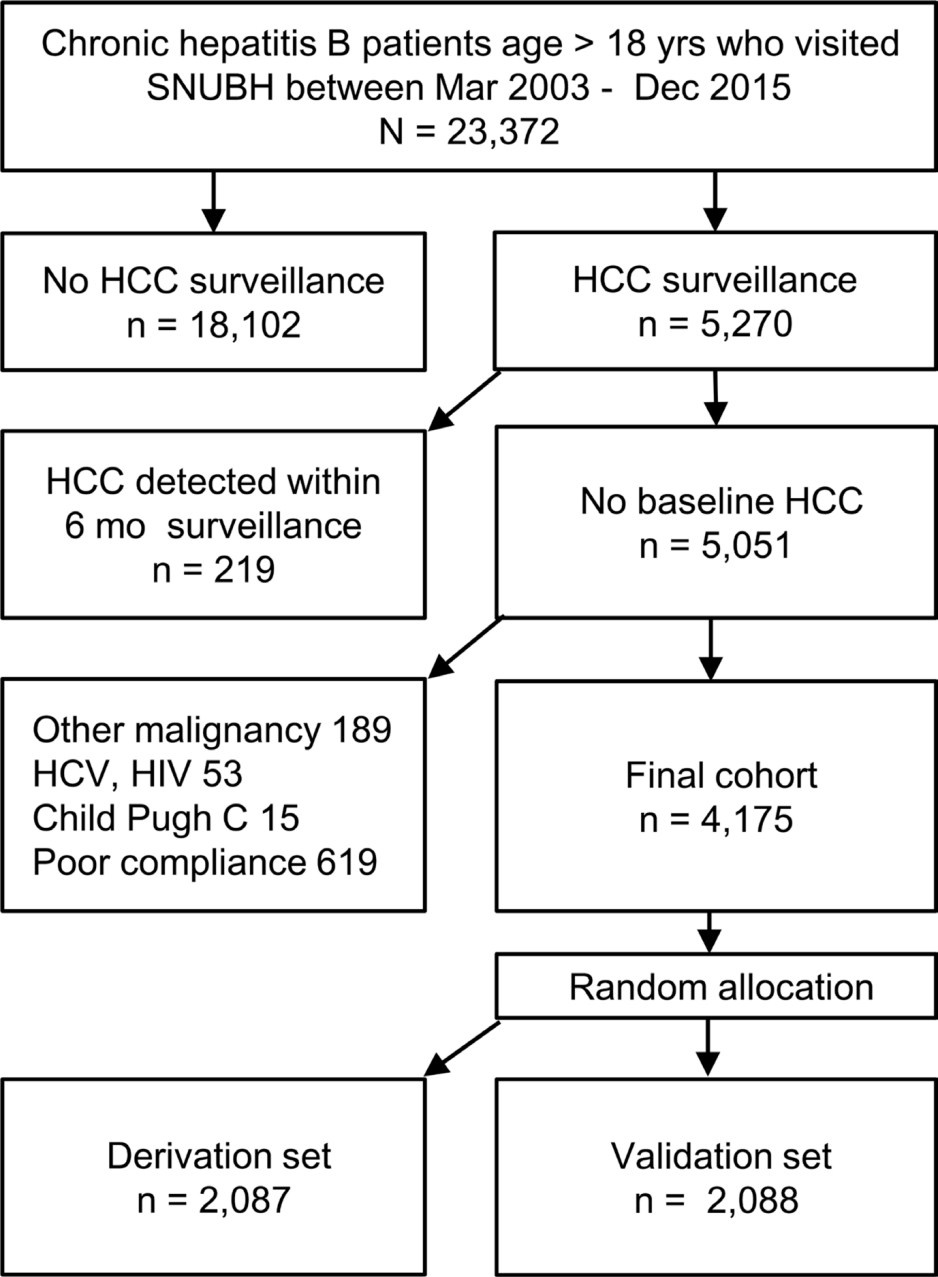
Participant flow diagram

The final cohort included 4,175 CHB patients, who were randomly allocated to the derivation set (*n* = 2,087) and the validation set (*n =* 2,088) (Figure 1). The characteristics of the two groups were similar at baseline and end of follow-up (Table 1). HCC developed in 222 patients with the incidence 0.769 per 1000 person-year during the median follow-up of 63 months (95% CI: 0.674–0.877). The HCC incidence was similar between the derivation and validation sets (Supplementary Figure 1A). Patients with cirrhosis had significantly higher HCC incidence compared to non-cirrhotic patients (2.94 vs. 0.17 per 1000 person-year, respectively, *p* < 0.001; Supplementary Figure 1B). The stage of HCC was BCLC 0, A, B and C for 41, 43, 5.3 and 10.7% of cases, respectively. The median size of the largest nodule was 2.0 cm (IQR, 1.6 cm). The main reason for advanced stage was involvement of portal vein: 83% of BCLC-C patients showed portal vein invasion.

**Table 1:** Characteristics of patients

Parameter	Baseline			End of follow-up		
	Derivation set	Validation set	*P* value	Derivation set	Validation set	*P* value
Number of patients	2,087	2,088	-	2,087	2,088	-
Follow-up, months	-	-	-	62 (76)	63 (74)	0.60
Nucleos(t)ide analog (%)	644 (31)	631 (30)	0.66	1,059 (51)	1,053 (50)	0.84
Age, years	45 (16)	45 (13)	0.13	52 (17)	52 (16)	0.12
Male (%)	1,202 (58)	1,285 (60)	0.12			
Liver cirrhosis (%)	431 (21)	446 (21)	0.59			
HCC development (%)				113 (5.4)	109 (5.2)	0.79
HBeAg positivity (%)	719 (34)	732 (35)	0.68	400 (19)	429 (21)	0.12
HBs Ag (IU/mL)	3831 (4086)	3832 (3878)	0.92	3577 (3386)	3529 (3135)	0.68
HBV DNA (log IU/mL)	3.7 (3.9)	3.6 (3.8)	0.34	1.8 (1.5)	1.8 (1.6)	0.10
Albumin (g/dL)	4.3 (0.4)	4.3 (0.4)	0.69	4.4 (0.4)	4.5 (0.3)	0.95
Bilirubin (mg/dL)	0.9 (0.5)	0.9 (0.5)	0.60	0.8 (0.5)	0.8 (0.4)	0.52
AST (IU/L)	30 (24)	30 (23)	0.96	25 (10)	25 (11)	0.26
ALT (IU/L)	35 (39)	35 (38)	0.29	24 (17)	24 (16)	0.05
Platelet (× 10^9^/L)	191 (79)	192 (75)	0.76	200 (78)	201 (77)	0.65
Prothrombin time (INR)	1.0 (0.1)	1.0 (0.1)	0.95	1.0 (0.1)	1.0 (0.1)	0.96

Data are presented as median (interquartile range) or numbers (percent)

ALT, alanine aminotransferase; AST, aspartate aminotransferase; HCC, hepatocellular carcinoma; INR, international normalization ratio.

### Age, sex, cirrhosis and serum AFP as independent predictors for presence of HCC

During the study period, 27,855 screening US tests were performed. After excluding 133 tests with ‘ambiguous’ association with HCC as described in the Methods, the remaining 13,908 and 13,814 tests from derivation and validation set were analyzed respectively. Logistic regression analysis of the 13,908 screening events in the derivation dataset identified factors predicting presence of HCC: new nodule(s) by US, old age, male sex, presence of cirrhosis, high AFP levels, high HBsAg titers, low albumin levels, high bilirubin levels, high AST levels, low platelet counts and prolonged prothrombin time were significant predictors of HCC presence (Table 2). Multivariate analysis selected old age, male sex, presence of cirrhosis and high AFP as independent predictors of HCC presence in addition to the positive US findings. Reclassification analysis also showed significant improvements in prediction of HCC presence by adding the four independent predictors to US-only screening, regardless of the stages of HCC (Table 3): the NRI of 1.31 and 1.29 for derivation and validation set, respectively, indicated about 65% (1/2(*NRI*) improvement in correct reclassification by the nomogram [21]. The positive IDIs also represented improved integrated difference in the corresponding Youden’s indices by the nomogram [22].

**Table 2:** Logistic regression analysis of predictors for presence of HCC

*n* = 13,908 Parameter	Univariate		Multivariate	
	OR (95% CI)	*P* value	OR (95% CI)	*P* value
Positive US finding ^a^	101.1 (61.1–167.1)	<0.001	38.5 (10.4–142.1)	**<0.001**
Age (years)	1.1 (1.0–1.1)	<0.001	1.1 (1.0–1.1)	**0.003**
Male sex	2. 0 (1.2–3.5)	<0.001	5.2 (1.2–22.4)	**0.03**
Liver cirrhosis	13.4 (7.4–24.7)	<0.001	7.2 (1.5–33.8)	**0.012**
AFP (Log ng/mL)	7.6 (5.8–10.1)	<0.001	19.4 (7.8–48.6)	**<0.001**
HBeAg positivity	1.0 (0.6–1.7)	0.90		
HBsAg titer (log IU/mL)	1.9 (1.0–3.5)	0.048	3.0 (0.9–9.7)	0.07
HBV DNA (log IU/mL)	1.0 (0.9–1.2)	0.81		
Nucleos(t)ide analog ^b^	1.7 (1.0–2.9)	0.04	0.5 (0.1–1.8)	0.26
Albumin (g/dL)	0.2 (0.1–0.3)	<0.001	1.7 (0.4–6.6)	0.45
Bilirubin (mg/dL)	1.2 (1.1–1.4)	0.006	0.6 (0.3–1.5)	0.30
AST >40 IU/L	4.4 (2.8–7.1)	<0.001	1.0 (1.0–1.0)	0.36
ALT >40 IU/L	1.6 (1.0–2.6)	0.06		
Platelet (10^9^/L)	1.0 (0.9–1.0)	<0.001	1.0 (0.02–1.0)	0.07
Prothrombin time (INR)	4.0 (1.7–9.2)	0.001	0.7 (0.004–23.0)	0.85

Numbers in parenthesis indicate 95% CI obtained from 1000 bootstrapping iterations.

^a^Detection of nodule(s) >1 cm which had not been previously characterized or showed changes in size or echo pattern.

^b^Exposure to nucleos(t)ide analog during study period.

AFP, alpha-fetoprotein; AST, aspartate aminotransaminase; ALT, alanine aminotransferase; INR. international normalized ratio; OR, odds ratio.

**Table 3:** Reclassification, sensitivity and specificity of HCC screening models

	Derivation set (*N* = 13,908)		Validation set (*N* = 13, 814)	
	US-only	HCC nomogram	US-only	HCC nomogram
All HCC				
NRI ^*a*^	-	1.31 (1.17–1.52)	-	1.29 (1.05–1.49)
IDI ^*a*^	-	0.14 (0.09–0.21)	-	0.13 (0.07–0.19)
Sensitivity ^*b*^	47.1 (35.1–59.4)	78.6 (67.1–87.5)	39.0 (26.5–52.6)	67.8 (54.4–79.4)
Specificity ^*b*^	99.1 (99.0–99.3)	96.1 (95.7–96.4)	99.2 (99.1–99.4)	95.9 (95.6–96.2)
Youden index	0.463 (0.349–0.577)	0.784 (0.703–0.829)	0.382 (0.263–0.501)	0.745 (0.637–0.815)
BCLC 0/A HCC				
NRI ^*a*^	-	1.29 (1.05–1.49)	-	1.14 (0.90–1.39)
IDI ^*a*^	-	0.13 (0.07–0.19)	-	0.07 (0.02–0.13)
Sensitivity ^*b*^	47.1 (35.1–59.4)	62.9 (50.5–75.4)	39.1 (25.1–54.6)	54.2 (40.8–67.3)
Specificity ^*b*^	99.1 (99.0–99.3)	98.7 (98.5–98.9)	99.3 (99.1–99.4)	98.7 (98.5–98.9)
Youden index	0.463 (0.349–0.577)	0.777 (0.707–0.812)	0.384 (0.263–0.501)	0.741 (0.624–0.812)

*N* indicates the numbers of HCC screening tests performed during the study period; numbers in parenthesis indicate 95% confidence intervals.

^a^Improvements by adding age, sex, cirrhosis and AFP levels to US by continuous net reclassification improvement (NRI) and integrated discrimination improvement (IDI) analyses with 300 bootstrapping iterations.

^b^Confidence interval from 1000 bootstrapping iterations; cut-off value of 140 for HCC nomogram score.

### Development of a nomogram for predicting presence of HCC

**Figure 2:**
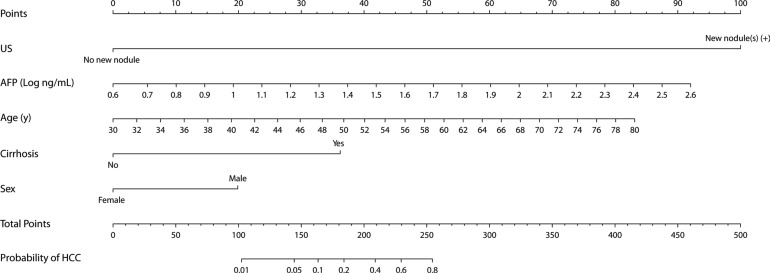
Nomogram for predicting presence of HCC in chronic hepatitis B patients on surveillance The individual point score for each variable is obtained on the corresponding perpendicular position on the top “Points” axis. Continuous values, i.e. age and logAFP, outside of the boundaries are replaced by the corresponding boundary value. The sum of all points, HCC nomogram scores, are converted to predicted HCC probability on the bottom probability axis.

**Figure 3:**
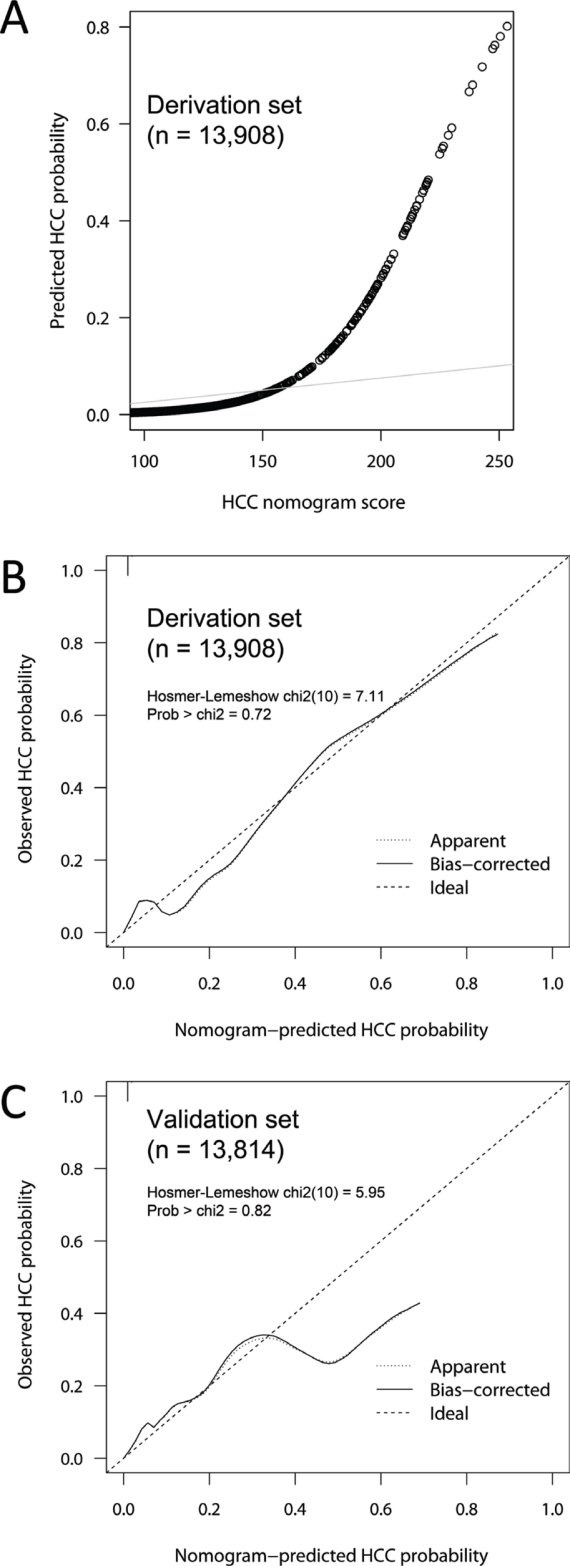
Calibration of the HCC nomogram score model The predicted probability of HCC presence was plotted against HCC nomogram score in the derivation dataset (**A**) Agreement between the predicted and observed HCC probabilities were plotted for the derivation (**B**) and validation (**C**) datasets with 300 bootstraps. The HCC nomogram score showed good calibration within the expected probability range up to 0.3, which corresponds to HCC nomogram score of 195. Hosmer and Lemeshowʼs goodness-of-fit test showed no significant discrepancies between the predicted and observed probabilities for HCC presence ( *p* = 0.72 and 0.82 for derivation and validation set, respectively).

Since the logistic analysis and reclassification analysis showed that traditional risk factors for HCC (age, sex, cirrhosis and AFP) provided additional information on the prediction of HCC presence, a nomogram was developed using the independent predictors to generate a combined indicator for estimating the probability of HCC presence (Figure 2). Calibration analysis showed that the HCC nomogram had good correlation between the predicted and observed probabilities within the clinically useful range (0–0.3), beyond which the model overestimated the probability in the validation set (Figure 3). The overall goodness-of-fit test showed that the nomogram satisfactorily fitted the observed probabilities without significant deviation ( *p* = 0.72 and 0.82 for derivation and validation set, respectively, by Hosmer-Lemeshow test).

### Performance of HCC nomogram: comparison of screening accuracy with US-only screening

We then sought to determine whether the nomogram outperformed US-only screening strategy in predicting presence of HCC. The sensitivity and specificity of US was 47.1% and 99.1% in the derivation cohort, respectively, and 39.1% and 99.3% in the validation cohort, respectively: among the 222 patients who developed HCC, 73 cases were confirmed by CT or MR although screening US showed no evidence of new nodule(s). Compared to US-only screening, the HCC nomogram showed higher sensitivity with minimal trade-off of specificity (Table 3): at the cut-off of 140, the HCC nomogram score had sensitivity and specificity of 62.9% and 98.7% in the derivation set, respectively, and 54.2% and 98.7% in the validation set, respectively. In cases with negative US tests, the nomogram showed 58–65% sensitivity at the specificity of 95% with the cut-off of 110–112 (Supplementary Table 1). ROC analysis demonstrated that the *C*-statistic value for the nomogram was significantly higher compared to US: 0.960 vs. 0.731 ( *p* < 0.001), respectively, in the derivation dataset, and 0.935 vs. 0.691, respectively, in the validation dataset ( *p* < 0.001) (Table 4). The superiority of HCC nomogram was significant regardless of tumor stages and history of nucleos(t)ide analog therapy.

**Table 4:** Comparison of areas under receiver operating characteristic curves between US- and HCC nomogram-based HCC screening

	Derivation set				Validation set				
All HCC	*N*	US-only	HCC nomogram	*P* value	*N*		US-only	HCC nomogram	*P* value
Total	13,908	0.731 (0.724–0.739)	0.960 (0.956–0.963)	<0.001	13, 814		0.691 (0.683–0.699)	0.935 (0.931–0.939)	<0.001
NA (+)	8,208	0.725 (0.715–0.735)	0.943 (0.938–0.948)	<0.001	7,997		0.698 (0.687–0.708)	0.913 (0.907–0.919)	<0.001
NA (–)	5,700	0.746 (0.735–0.758)	0.985 (0.981–0.988)	<0.001	5,817		0.674 (0.662–0.686)	0.967 (0.963–0.972)	<0.001

BCLC 0/A	*N*	US-only	HCC nomogram	*P* value	*N*	US-only		HCC nomogram	*P* value
Total	13,833	0.741 (0.734–0.749)	0.956 (0.952–0.959)	<0.001	13,717	0.692 (0.684–0.700)		0.943 (0.939– 0.947)	<0.001
NA (+)	8,147	0.721 (0.711–0.731)	0.938 (0.933–0.943)	<0.001	7,907	0.702 (0.692–0.712)		0.925 (0.919–0.931)	<0.001
NA (–)	5,686	0.791 (0.780–0.801)	0.983 (0.979–0.986)	<0.001	5,810	0.674 (0.662–0.686)		0.968 (0.963–0.972)	<0.001

*N* indicates the number of HCC screening tests performed during the study period.

The numbers in parenthesis indicate 95% confidence intervals.

NA (+) and NA (–) denote patients with or without exposure to nucleos(t)ide analog therapy during the study period.

All HCC: total patients; BCLC 0/A: patients without development of advanced HCC, i.e. BCLC B/C/D.

### Cost-effectiveness of HCC nomogram-guided screening model: decision curve analysis

**Figure 4:**
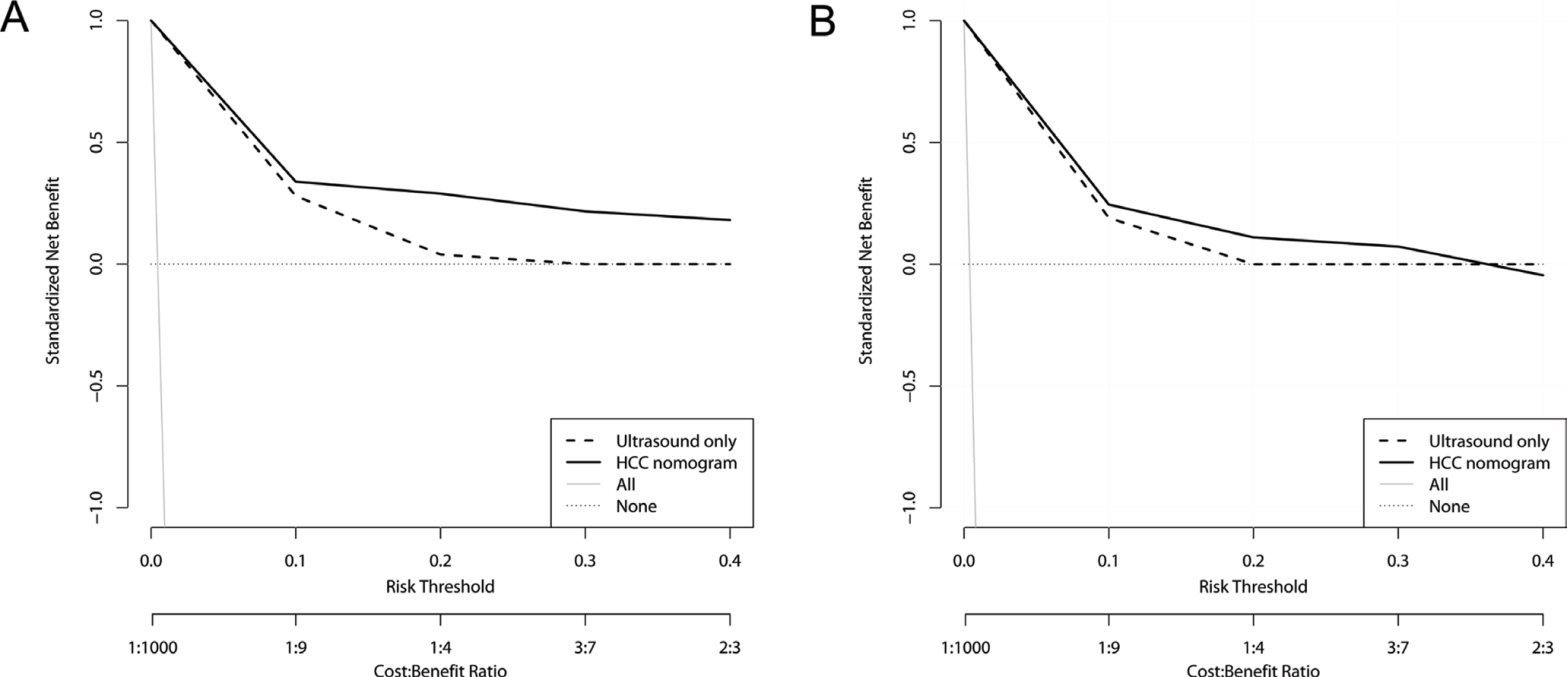
Decision curve analysis for HCC screening models Decision curves for derivation set (**A**) and validation set (**B**). Ultrasound-only indicates traditional US-based screening in which decisions to request confirmatory tests are guided only by positive US tests. *HCC nomogram* indicates that the decisions are guided by HCC nomogram scores. Risk threshold and cost:benefit ratio indicate the relative significance of correct detection of HCC to correct exclusion of HCC of the models. *All* indicates that all CHB patients receive confirmatory tests, i.e. dynamic imaging studies or biopsy, and *None* indicates that no patients receive confirmatory tests. The net benefit of HCC nomogram was higher than that of US-only across given range of threshold probabilities, indicating that nomogram-based screening model would produce cost-effective clinical outcome irrespective of patient preference.

Because the increased sensitivity of HCC nomogram was associated with small decrease in specificity, decision curve analysis was performed to determine whether the benefit of the nomogram was clinically useful in terms of cost-effectiveness. HCC nomogram-guided screening model had greater net benefit compared to the US-only screening in the risk threshold range between 0 and 0.3, both in the derivation and validation dataset (Figure 4). This result suggested that HCC nomogram-guided decision to perform confirmatory tests may be clinically cost-effective for patients whose cost-benefit ratio, i.e., harms of confirmatory tests to harms of missing HCC, is less than 2/3.

## DISCUSSION

In this study, we demonstrated that consideration of individual risks helps predicting presence of HCC in a real-world CHB cohort on surveillance: the HCC nomogram which integrated the HCC risk predictors with the results of screening US had superior accuracy compared to US-only screening. The nomogram-guided screening model also showed cost-effectiveness by decision curve analysis.

The reported sensitivity of US for early HCC vary among studies [23], but recent studies suggested relatively low sensitivity in the surveillance setting [7, 8, 24]. Our data also revealed similarly low sensitivity of US in CHB. Advances in the dynamic imaging technology allows imaging diagnosis of smaller HCCs [2, 5], and it can be speculated that the sensitivity of US may decrease as HCC is diagnosed in the earlier stage [9]. In addition, we classified serial screening data up to 6 months before final diagnosis of HCC as HCC-associated US, and this classification scheme may also have contributed to the low sensitivity.

Several studies have shown that age, sex, cirrhosis and AFP levels are predictors for long-term HCC risk in CHB [14–20, 25, 26]. A recent Korean multicenter cohort study also confirmed the long-term predictive significance of age, sex and cirrhosis in CHB patients on oral NA therapy [26]. Our data demonstrated that these predictors can be used to estimate the probability of *presence* of HCC as well. This finding is in line with the recent report in which age, AFP, platelets, and alanine aminotransferase (ALT) predicted immediate development of HCC [20]. The unique strength of our study was the inclusion of US test results in the multivariate analysis, which suggested that the four predictors may supplement US-based HCC screening. Since US is the only recommended screening tool for HCC, confirmatory dynamic imaging studies are triggered only by positive US findings and underlying risks of HCC are not reflected in the clinical decisions once surveillance is started under current recommendations [2, 3]. Our HCC nomogram showed good calibration profile in predicting presence of HCC within the clinically relevant range, beyond which the confirmatory tests would be warranted without considering the nomogram scores. Furthermore, the HCC nomogram had significantly superior reclassification and discrimination characteristics over US-only screening, regardless of HCC stages. Taken together, it can be suggested that consideration of age, sex, status of cirrhosis and AFP levels along with US results improves the screening accuracy of HCC detection, probably by identifying additional patients for whom dynamic imaging studies are likely to produce positive results [27]. Long-term prospective validation is needed, however, to determine whether personalized surveillance based on HCC nomogram improves the performance and outcome of HCC surveillance in CHB.

Current AASLD and EASL guidelines do not recommend AFP as a screening test, mainly because of low sensitivity and specificity [2, 3, 28, 29]. Hepatitis activity may elevate AFP levels in CHB [30, 31], and elevated AFP levels may just signify increased risk for future development of HCC [18, 20, 29, 32]. However, AFP comprised one of the major components of our nomogram. Hepatitis activity may decrease over the course of CHB, either with or without NA therapy, and false positivity of AFP may decrease accordingly [33]. In our cohort, the median ALT level was 24 IU/L with IQR of 17 at the end of follow-up, and HBV DNA levels also showed decreasing tendency. Patients with hepatitis flare were likely to start NA therapy during the study period, as suggested in Table 1. Because we analyzed all of the serial screening data rather than baseline or final ones, AFP values associated with hepatitis flare may not have significantly affected the performance of the nomogram. This explanation is also in line with the finding that the superiority of nomogram over US was independent of exposure to NA therapy. The fact that elevated AFP may imply both high baseline risk and presence of HCC may not necessarily disprove its role, but rather render AFP suitable for our probability-oriented nomogram.

Currently AFP is seldom used alone in HCC surveillance, and there have been several reports indicating increase in the sensitivity of US-based surveillance by adding AFP [7, 34]. However, previous cost analyses provide no solid evidence supporting the combined use of AFP, and the benefit may be offset by increased false positivity and the following costs of confirmatory tests [7, 11, 34–36]. To address the same issue in our data, we performed the decision curve analysis, which revealed that the HCC nomogram-based screening model had greater net benefit compared to US-only model in the clinically appropriate risk threshold range [37], indicating that HCC nomogram-guided screening model may be cost-effective and can be recommended for all CHB patients with reasonable threshold probabilities without compromising specificity [38].

The main limitation of our study is retrospective design in single center. Consecutive patient was recruited from the comprehensive electronic registry [39–41] to reduce selection bias, and we adopted a split-sample approach with bootstrapping to reduce the possibility of overfitting. Nevertheless, our findings need further external validation in prospective settings. Secondly, our simulated decision curve analysis needs to be validated by including the performances of confirmatory imaging tests and cost-effectiveness parameters which are specific to regional settings. Lastly, the final outcome measurements of prospective validation studies need to include improvement of survival.

In conclusion, a nomogram composed of age, sex, presence of cirrhosis, serum AFP levels and US findings better predicts the probability of presence of HCC compared to US-only screening in CHB on surveillance. HCC nomogram-based screening has superior performance compared to US-only screening and cost-effective. The clinical usefulness of HCC nomogram-guided surveillance strategy needs to be validated in prospective studies.

## MATERIALS AND METHODS

### Study population

This single center retrospective cohort study enrolled consecutive CHB patients who were over age eighteen and underwent regular surveillance for HCC between Mar. 2003 and Dec. 2015 in a tertiary referral center in South Korea. Clinical and laboratory data were retrieved from the liver disease registry of Seoul National University Bundang Hospital Clinical Data Warehouse [40, 42]. Patients with detection of HCC within 6 months after initial screening, malignancy other than HCC, hepatitis C virus or human immunodeficiency virus coinfection, Child-Pugh class C or non-compliance of surveillance were excluded (Figure 1).

Screening US examination was carried out every 6–12 months along with blood tests including serum AFP levels, transaminases, prothrombin time, albumin, bilirubin and platelet counts. The presence of liver cirrhosis was defined by ultrasonographic features (coarse liver echotexture with nodularity) plus evidence of portal hypertension including ascites, splenomegaly, thrombocytopenia (<100 × 10^9^/L) and varices [15, 43]. US findings were classified dichotomously as “positive” if nodule(s) >1 cm was detected which had not been previously characterized or showed changes in size or echo pattern [2], or “no evidence of HCC” otherwise. When US detected suspicious nodule(s), multidetector 4-phase CT or MRI was performed. Dynamic imaging studies were also considered at the attending physicians’ discretion in the case of successive rise in serum AFP levels, incomplete US examination due to poor sonic window, or very coarse echo-pattern with numerous nodules [3, 11]. Diagnosis of HCC was made according to the AASLD criteria [2].

The institutional review board of our hospital approved this study (IRB No: B-1609/361102). All clinical investigation has been conducted according to the principles expressed in the Declaration of Helsinki. Informed consent was wavered by IRB, due to the retrospective observational nature of study and anonymous analysis of data.

### Data collection and imputation

In patients who developed HCC, the US examinations up to 6 months before the confirmatory test were classified as “associated with HCC” [20], whereas US examinations performed more than 12 months before the confirmatory test were classified as “not associated with HCC” and analyzed as such. US examinations 6–12 months before confirmatory test were classified as “ambiguous” and excluded from the logistic regression analysis. Blood tests performed within 45 days before or 15 days after each US examination were linked to the corresponding US results and data outside of this range were excluded. Missing AFP data were predicted by multiple imputations with the number of imputations of 10 using bootstrap and predictive mean matching by R *aregImpute* package. The variables included in the multiple imputation model were previously identified as significant predictors of AFP elevation: presence of cirrhosis, AST, ALT, albumin, prothrombin time, HBV DNA levels and history of nucleos(t)ide analogues [41]. HCC was excluded from the imputation model, however, for the conservative estimation of the predictive role of AFP.

### Development and validation of a model predicting presence of HCC

Logistic regression analysis was used to identify predictors for presence of HCC at the time of each US measurement. For the internal validation, the final patients were randomly allocated to either derivation or validation dataset (Figure 1), and bootstrapping procedure was performed [44]. Predictor variables were selected by backward stepwise selection with a *P* value > 0.05 for removal. The outcome variable of the logistic model was presence of HCC. Reclassification analysis was performed by continuous net reclassification improvement (NRI) and integrated discrimination improvement (IDI) index to determine whether addition of independent predictors improve the prediction of present HCC [21, 22].

A nomogram was established from the independent predictors of the logistic model. The endpoint of the nomogram was detection of HCC at the time of US examination. The HCC nomogram score, the point sum of each parameter, was tested for calibration and discrimination [22, 45]. Calibration of the nomogram was evaluated for correct detection of HCC by calibration curves and Hosmer-Lemeshow goodness-of-fit test [46]. The discriminative ability was assessed by the receiver operating characteristic (ROC) curve analysis with comparison of concordance (*C*) statistic [47].

### Decision curve analysis

In order to determine whether HCC nomogram is cost-effective compared to US-only screening, we employed decision curve analysis. Decision curve analysis takes misclassification costs of diagnostic tests into account without assuming pre-defined utility, and balances the benefit of true positivity against the cost of false positivity of diagnostic tests [37, 38]. The threshold probability is defined as the probability where the expected benefit of opting for confirmatory tests is equal to the expected benefit of avoiding confirmatory tests [38, 47]. For example, the risk threshold of 0.1 corresponds to cost-benefit ratio of 1:9 [0.1 / (1–0.1)] and signifies that a rational patient with this risk threshold will opt for HCC nomogram-based screening instead of US-only screening if the expected probability of having HCC is 0.1 or greater because the harms associated with a missed HCC is nine times greater than the harms associated with unnecessary additional tests. *Net benefit* indicates the difference between proportions of true positive and false positive, weighted by the risk threshold [38].

### Statistical analysis

Statistical analyses performed using STATA version 14 (College Station, Texas) and R package (version 3.3.2, www.r-project.org). Continuous and categorical variables were tested by Student’s *t*-test and χ^2^ test, respectively. Kaplan-Meier analysis was used to calculate the cumulative incidence of HCC of the study population. Generation of nomogram and calibration analysis was performed by R *rms* package. Comparison of ROC curves was made by STATA *ROCgold* command. Reclassification analysis was performed by STATA *incrisk* command. Decision curve analysis was performed by R *DecisionCurve* package.

## SUPPLEMENTARY MATERIALS FIGURE AND TABLE



## Abbreviations

HCC: hepatocellular carcinoma
CHB: chronic hepatitis B
US: ultrasonography
CT: computed tomography
MRI: magnetic resonance imaging
HBV: hepatitis B virus
AFP: alpha-fetoprotein
NRI: net reclassification improvement
IDI: integrated discrimination improvement
ROC: receiver operating characteristic
ALT: alanine aminotransferase
