# Supplementary material for: Development of a nomogram for screening of hepatitis B virus-associated hepatocellular carcinoma
Source: Oncotarget. 2017 Nov 18;8(63):106499–510. doi: 10.18632/oncotarget.22498 (PMC5739751; doi:10.18632/oncotarget.22498)
Supplement: Supplementary file 1 [file oncotarget-08-106499-s001.pdf]

Development of a nomogram for screening of hepatitis B virus-associated hepatocellular carcinoma

SUPPLEMENTARY MATERIALS

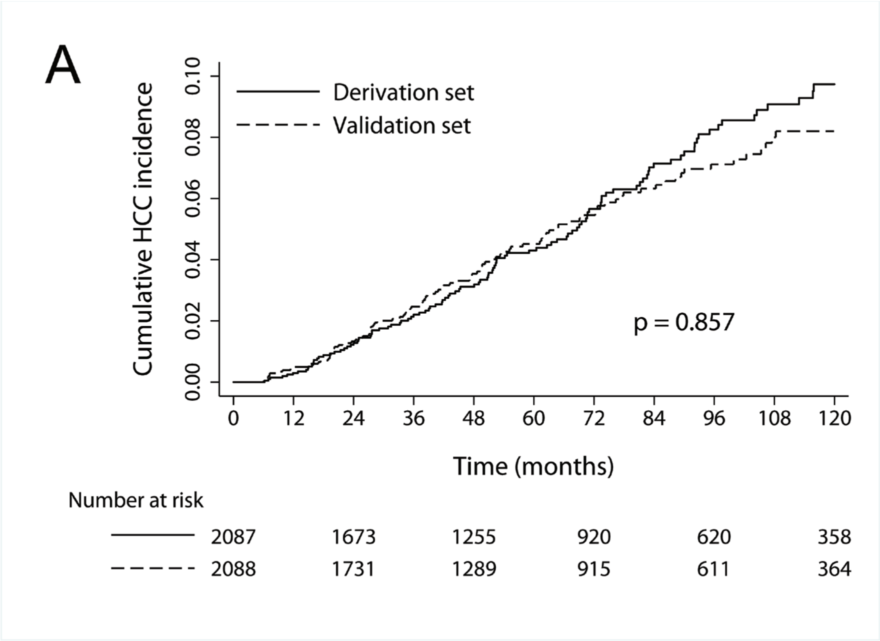

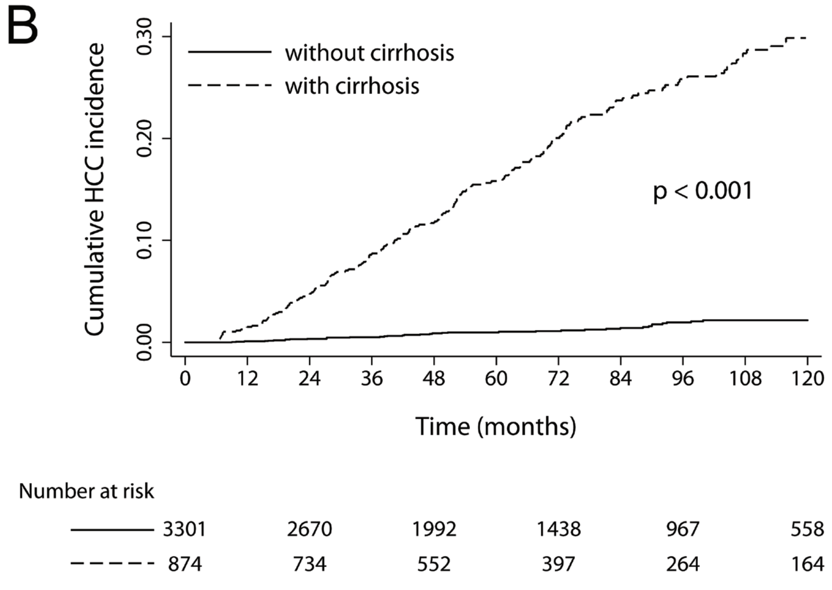

Supplementary Figure 1: Incidence of HCC in the study cohort. (**A**) Comparison between derivation and validation dataset. (**B**) Comparison between patients with or without liver cirrhosis.

Supplementary Table 1: Sensitivity and specificity of nomogram score by US positivity

**Derivation group**				
**Specificity**	**US positive (*n* = 154)**		**US negative (*n* = 13,754)**	
	**Sensitivity (95% CI)**	**Nomogram score**	**Sensitivity (95% CI)**	**Nomogram score**
95	45.5 (28.1–63.6)	220	64.9 (47.5–79.8)	110
97	33.3 (18.0–51.8)	229	59.5 (42.1–75.2)	117
99	3.0 (0.08–15.8)	271	43.2 (27.1–60.5)	132
**Validation group**				
**Specificity**	**US positive (*n* = 129)**		**US negative (*n* = 13,685)**	
	**Sensitivity (95% CI)**	**Nomogram score**	**Sensitivity (95% CI)**	**Nomogram score**
95	13.04 (2.8–33.6)	233	58.3 (40.8–74.5)	112
97	13.04 (2.8–33.6)	233	47.2 (30.4–64.5)	116
99	13.04 (2.8–33.6)	233	36.1 (20.8–53.8)	132
